# The impact of parenting stress on parents of school-age children with drug-resistant epilepsy

**DOI:** 10.3389/fped.2022.948286

**Published:** 2022-09-21

**Authors:** Hsin-Hui Lu, Chun-Yu Tsai, I-Ching Chou, Jeng-Dau Tsai

**Affiliations:** ^1^Department of Psychology, Chung Shan Medical University, Taichung, Taiwan; ^2^Clinical Psychological Room, Chung Shan Medical University Hospital, Taichung, Taiwan; ^3^Division of Pediatrics Neurology, China Medical University Children’s Hospital, Taichung, Taiwan; ^4^Graduate Institute of Integrated Medicine, China Medical University, Taichung, Taiwan; ^5^Department of Pediatrics, Chung Shan Medical University Hospital, Taichung, Taiwan; ^6^School of Medicine, Chung Shan Medical University, Taichung, Taiwan

**Keywords:** parenting stress index, school age, drug-resistant epilepsy, epilepsy-specific stress, cognitive dysfunction

## Abstract

**Background:**

Psychological burdens can affect the quality of life among parents of children with epilepsy, especially parents of children with poor seizure control. The impact of stress on the parents of children with epilepsy is significantly comorbid with their children’s cognitive dysfunction and the severity of epilepsy. The aim of this study was to assess the stress levels of parents of school-age children with drug-resistant epilepsy (DRE) and controlled-epilepsy after considering the children’s cognitive ability.

**Methods:**

The study participants consisted of 35 children with typical development in the control group, 25 in the controlled-epilepsy group, 26 in the DRE group, and their parents. We used the Chinese version of the Parenting Stress Index (PSI) to measure the stress levels of all parents; and the Wechsler intelligence scale for children-fourth edition (WISC-IV) Chinese version to assess the children’s cognition levels.

**Results:**

Parenting stress was significantly higher among the parents of children with DRE than of those in the control and controlled-epilepsy group. The PSI’s child domain showed statistically significant subscales of adaptability, acceptability, demandingness, and distractibility/hyperactivity. Moreover, the high-risk ratio on the acceptability, adaptability, demandingness, and distractibility/hyperactivity subscales were also higher for the DRE group than for the controlled-epilepsy group and for the control group.

**Conclusion:**

Seizure severity significantly influences parenting stress after considering cognitive dysfunction in children with epilepsy. Therefore, pediatricians and clinicians should consider epilepsy-specific stress in parents of children with DRE.

## Introduction

Seizures affect the daily lives of individuals with epilepsy and their caregivers. The manifestations and consequences of seizures can affect the physical development of such individuals and cause a psychological burden, especially when the seizures disrupt the developmental period essential to cognition and social ability (1). Children with epilepsy have mental health condition (i.e., emotional and behavioral problems) more often than the general population (2), and the resulting psychological burden of epilepsy can affect the quality of life of children, particularly those with poor seizure control (3). Childhood drug-resistant epilepsy (DRE) is also often comorbid with cognitive dysfunction, potentially restricting the daily activities of affected individuals and their caregivers (2). Therefore, the caregivers of children with epilepsy, particularly DRE, frequently experience significant stress (4, 5).

Stress is a normal part of life, and stress-response systems (e.g., emotional reactivity, vigilance, physiological arousal, etc.) alert individuals to act and react. The parental role always involves some level of stress. Parenting stress arises from coping with the day-to-day challenges of raising a child and balancing the demands of the caregiver role with the available resources. When those challenges include children’s health issues, it is essential to understand parental psychological burden (6). Specifically, the stress associated with caring for children affected by seizures ticks all the boxes for the traumatic stress caused by severe, recurring, chronic, and unpredictable illnesses. In addition, child illness uncertainty has been proven to moderate the relationship between parents’ psychological distress and child-reported depressive moods among children with chronic illness (7). It may also affect children’s health outcomes if it interferes with disease management (8).

Overall, the impact of stress on the parents of children with DRE is significantly comorbid with their children’s cognitive dysfunction and the severity of epilepsy (9). However, whether the parents of children with DRE experience higher levels of parenting stress related to their children’s epilepsy-specific characteristics was unclear. This study aimed to assess the relative parenting stress levels of parents of school-age children with typical development (controls), controlled epilepsy (CE), and DRE. Considering the children’s cognitive function, the characteristics of children having severe, recurring, chronic, and unpredictable illnesses had been found to increase the parenting stress. Therefore, we hypothesized that the parents of children with DRE had sill higher stress than children with controlled epilepsy after considering their cognitive dysfunction.

## Materials and methods

This study used a cross-sectional design for school-age children.

### Participants

We provided the criteria for patient selection and exclusion before enrolling the study participants who had provided informed consent. The participants were 6–12-year-old children with a history of epilepsy who were receiving anticonvulsant treatment and their parents. Children with epilepsy that responds to anticonvulsants is defined as controlled-epilepsy (CE). Children with epilepsy that fails to respond after adequate trials of >2 tolerated and appropriately chosen and used anticonvulsant schedules is defined drug-resistant epilepsy (DRE) (10). The participants in the control group matched the CE and DRE groups in terms of age and sex and did not present any other neurological or psychological disorders.

### Measurements

#### Assessment of parenting stress

We used the Chinese version of the Parenting Stress Index (PSI) (11) to measure the parents’ stress levels. The PSI is a self-report instrument that assesses the stress levels of parents and caregivers in parent–child interactions. It scores participants’ responses on 13 subscales describing aspects of the child and parent domains, using the sum of the scores as a measure of the total stress in the parent–child interaction. The subscales on the child domain examine the children’s adaptability, acceptability, demandingness, mood, distractibility/hyperactivity, and parental reinforcement. The subscales of the parent domain examine how parents perceive themselves in their role and the impact of parenting on individual‘s life, such as depression, attachment (to the child), role restriction, competence, social isolation, spouse/parenting partner relationship, and health. We converted the raw PSI scores to percentile rank (PR) scores using norms published in Taiwan, under which the high risk of each subgroup was defined as PR ≥90, and the high-risk ratio (HRR) is defined as (number of PR ≥90)/(total numbers).

#### Assessment of cognitive dysfunction: Intelligence quotient levels

For the intelligence quotient (IQ) level, psychological assessments were conducted by using the Wechsler intelligence scale for children-fourth edition (WISC-IV) Chinese version (12). The levels are classified as ≧ 80, 70–79, 55–69, and 45–54. We further classify the IQ scores into two levels: ≥80 (the normal cognitive level) and <80 (the cognitive dysfunction level) in later analysis.

### Procedure

First, the researcher informed the participants and their parents of the research procedures, after which they provided informed consent. Next, all participants were administered WISC-IV; and all participants’ parents completed the PSI and provided demographic information. Data collection from participants and their parents were conducted in the two quiet rooms, respectively. The duration of test administration was 1.5–2 h.

### Statistical analysis

For our statistical analysis, we used IBM SPSS Statistics for Windows, Version 26.0 (Armonk, New York, USA: IBM Corp., Inc.). We performed analyses of covariance (ANCOVAs) to examine the differences among the three groups’ PSI scores with IQ level as a covariate and the Scheffe’s *post hoc* tests. Furthermore, analyses of variance (ANOVAs) to examine the differences among the three groups’ PSI high-risk ratio and the Scheffe’s *post hoc* tests. Effect sizes from the ANCOVAs/ANOVAs were calculated using partial eta square (η_*p*_^2^), which can be translated directly into percentage of variance explained. If the effect size of the main effect of group is large, it would detect such an effect in smaller sample numbers, whereas a smaller effect size would require larger sample sizes (13). Cohen (14) provided a basic framework for interpreting these effects as small (η_*p*_^2^ = 0.01), moderate (η_*p*_^2^ = 0.06), or large (η_*p*_^2^ = 0.15). We also used chi-square and Fisher’s exact tests to compare the group proportions with the qualitative data. The above tests adopted a significance level (α) of *p* < 0.05.

## Results

### Sample characteristics

**FIGURE 1:**
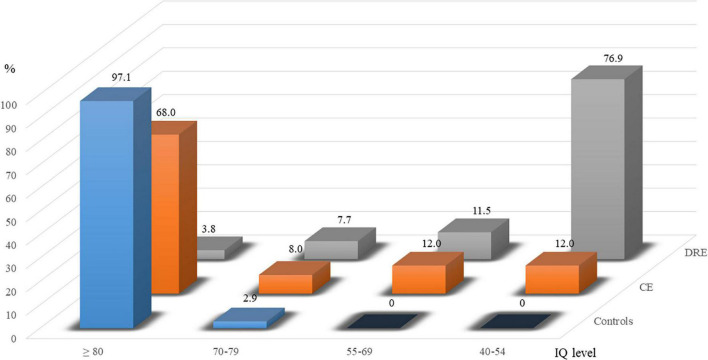
Percentages of ≧80, 70–79, 55–69, and 45–54 in groups of controls, controlled epilepsy (CE), and drug-resistant epilepsy (DRE).

Table 1 shows the demographic data of the study participants: 35 children in the control group (25 boys; 19 children >9 years); 25 in the CE group (19 boys; 13 children >9 years); and 26 in the DRE group (16 boys, 14 children >9 years). Sex (*p* > 0.05) and age (*p* > 0.05) are not significantly different among the groups. To compare the IQ level with the cut-off, point of 80, there is statistical difference among the three groups (*p* < 0.001). Subsequently the distributions of IQ levels are displayed in Figure 1. There are 97.1% and 68.0% ≧ 80 of IQ level in controls and CE group. In the DRE group, 76.9% are moderate intellectual disability (40–54 of IQ level).

**TABLE 1:** Demographic data of school-age children, including control, CE, and DRE groups, *N* = 86.

Groups	Control	CE	DRE	*p-*value
Numbers	35	25	26	
**Sex**				
Boy	25(71.4)	19(76.0)	16(61.5)	NS
Girl	10(28.6)	6(24.0)	10(38.5)	
**Age**				
≤9 years	19(54.3)	13(52.0)	10(38.5)	NS
>9 years	16(45.7)	12(48.0)	16(61.5)	
**IQ level**				
≥ 80	34(97.1)	17(68.0)	1(3.8)	<0.001
<80	1(2.9)	8(32.0)	25(96.2)	

Data are shown as n (%); IQ, intelligence quotient; CE, Controlled-epilepsy; DRE, Drug-resistant epilepsy. NS, Not significant.

### Raw score

**FIGURE 2:**
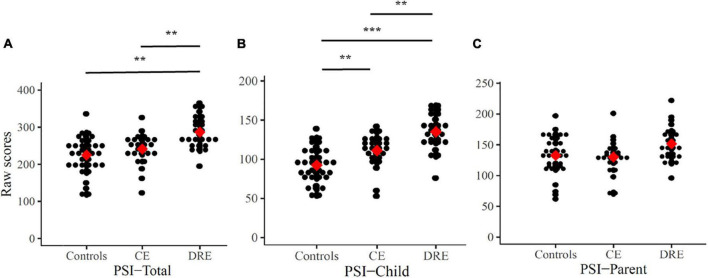
Raw scores of PSI-Total **(A)**, PSI-Child **(B)**, and PSI-Parent **(C)**. Mean are presented as red square. CE: controlled-epilepsy; DRE: drug-resistant epilepsy. The results of the Scheffe’s *post hoc* tests are marked; **p < 0.01 and ***p < 0.001.

The raw scores of PSI are (or not) adjusted with IQ level, and there are statistically significant differences (ANOVAs/ANCOVAs) in raw PSI-Total scores (*p* < 0.001, η_*p*_^2^ = 0.26) and raw PSI-Child scores (*p* < 0.01, η_*p*_^2^ = 0.13). In Figure 2, the results of the Scheffe’s *post hoc* tests in the PSI-Total scores show statistical significance in DRE vs. CE (*p* < 0.01) and DRE vs. controls (*p* < 0.01). In PSI-Child, it shows statistical significance in DRE vs. CE (*p* < 0.01), DRE vs. controls (*p* < 0.001) and CE vs. controls (*p* < 0.01). However, there is no significant difference in the raw score of the PSI-Parent.

Table 2 shows the results for the three groups. The results are adjusted with the level of IQ, and there are statistically significant in the domains of adaptability (*p* < 0.05, η_*p*_^2^ = 0.08), acceptability (*p* < 0.01, η_*p*_^2^ = 0.14), demandingness (*p* < 0.01, η_*p*_^2^ = 0.14), and distractibility/hyperactivity (*p* < 0.05, η_*p*_^2^ = 0.11).

**TABLE 2:** Raw scores of parental stress index in school-age children in control, CE, and DRE groups, *N* = 86.

Groups	Control	CE	DRE	Adjusted *p-*value^†^
Numbers	35	25	26	
**Child domain**				
Adaptability	21.80(7.38)	27.72(6.13)	33.58(7.81)	<0.05
Acceptability	14.09(4.02)	17.28(4.61)	23.88(4.47)	<0.01
Demandingness	20.71(5.51)	25.12(4.89)	29.85(5.05)	<0.01
Mood	10.57(3.65)	11.40(3.56)	13.23(3.98)	NS
Distractibility/hyperactivity	17.14(5.39)	21.40(5.45)	24.23(5.10)	<0.05
Reinforces parent	8.03(3.25)	8.12(3.02)	9.69(3.16)	NS
**Parents domain**				
Depression	23.51(6.86)	23.96(5.58)	26.77(6.59)	NS
Attachment to child	14.57(3.16)	13.56(3.63)	14.69(3.50)	NS
Role restriction	18.83(6.77)	17.24(5.80)	21.04(5.97)	NS
Competence	26.66(6.70)	28.48(6.32)	30.50(7.03)	NS
Social isolation	16.29(5.59)	16.16(4.69)	16.27(5.35)	NS
Spouse	22.00(6.66)	21.44(5.69)	22.35(5.82)	NS
Health	10.71(4.16)	9.24(2.67)	11.19(2.91)	NS

Data are shown as mean (standard deviation); †, ANCOVAs adjusted with IQ level. CE, Controlled-epilepsy; DRE, Drug-resistant epilepsy. NS, Not significant.

### Percentile rank scores

**FIGURE 3:**
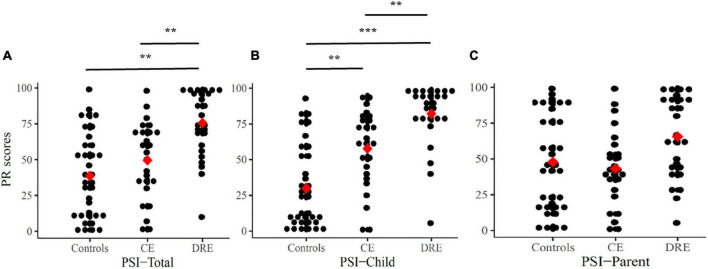
Percentile rank (PR) scores for PSI-Total **(A)**, PSI-Child **(B)**, and PSI-Parent **(C)**. Mean are presented as red square. CE, controlled-epilepsy; DRE, drug-resistant epilepsy. The results of the Scheffe’s *post hoc* tests are marked; **p < 0.01 and ***p < 0.001.

The PR of PSI are (or not) adjusted with the level of IQ, and there are statistically significant differences (ANOVAs/ANCOVAs) in the PSI-Total (*p* < 0.001, η_*p*_^2^ = 0.26) and PSI-Child (*p* < 0.01, η_*p*_^2^ = 0.13) among the three groups. In Figure 3, the results of the Scheffe’s *post hoc* tests in the PSI-Total scores show statistical significance in DRE vs. CE (*p* < 0.01) and DRE vs. controls (*p* < 0.01). In PSI-Child, it shows statistical significance in DRE vs. CE (*p* < 0.01), DRE vs. controls (*p* < 0.001) and CE vs. controls (*p* < 0.01). However, there is not any statistical significant difference in the PRs of the parent domain.

### High risk ratio

**FIGURE 4:**
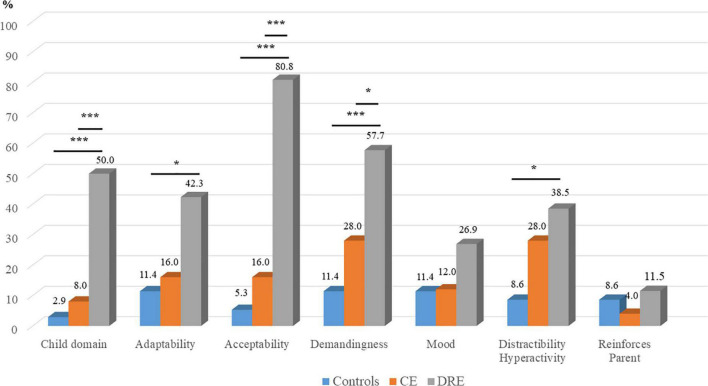
High risk ratios (HRR) of PSI-Child and subscales. CE, controlled-epilepsy; DRE, drug-resistant epilepsy. The results of the Scheffe’s *post hoc* tests are marked; *p < 0.05 and ***p < 0.001.

The HRRs of the PSI child domains are examined (ANOVAs), show statistically significant differences in adaptability (*p* < 0.05, η_*p*_^2^ = 0.11), acceptability (*p* < 0.001, η_*p*_^2^ = 0.50), demandingness (*p* < 0.001, η_*p*_^2^ = 0.18), and distractibility/hyperactivity (*p* < 0.05, η_*p*_^2^ = 0.09). In the PSI-Child (Figure 4), the *post hoc* tests show statistically significant in DRE vs CE (*p* < 0.001) and DRE vs. controls (*p* < 0.001). In the child domain of adaptability, there is statistical significance in DRE vs controls (*p* < 0.01). In acceptability, there is statistical significance in DRE vs CE (*p* < 0.001) and DRE vs. controls (*p* < 0.001). In demandingness, there are statistical significance in DRE vs. CE (*p* < 0.05) and DRE vs. controls (*p* < 0.001). In distractibility/hyperactivity, there is statistical significance in DRE vs. controls (*p* < 0.05).

## Discussion

Unlike previous large-scale studies (15, 16), this was a study with a small sample size. However, it used a rigorous matched pairs design following three groups of children, and the effect sizes were moderate to large. These factors increase the validity of this study’s findings. We found that the PSI-Total and PSI-Child raw scores were the highest for the children in the DRE group among the three groups. Moreover, the PSI-Child percentile rank scores were also higher for the children with DRE than for those with CE and those in the control group. These findings align with previous studies showing that parents of children with DRE experience significantly higher stress levels than the general population (17). We further found that the parents of children with DRE had higher scores for parenting stress than the parents of children with CE after considering their cognitive function. This indicates that children’s high-severity seizures hampered parental daily life and experienced increased parenting stress, which may limit the ability of parents of children with high-severity seizures to pursue interests outside the caregiving role.

Parenting stress has been identified as a crucial determinant of dysfunctional parenting, and raising children with DRE has been associated with markedly increased caregivers’ stress scores (18, 19). The elevated stress levels experienced by parents of children with DRE may be related to the presence of comorbid cognitive dysfunction in the children (20). We identified high levels of parenting stress in the parents of children with DRE in the child domain but not in the parent domain, showing that the increased stress was predominantly related to parenting and the characteristics of children with epilepsy. It reflected a possible transactional process between the relationships of caregivers’ psychological distress and children’s uncontrolled-epilepsy that is critical to explore and address clinically.

Seizures have a time-limited and reversible negative impact on cognition (i.e., ictal and postictal cognitive impairment); intermittent epileptic discharges may also affect cognitive performance (21). Previous studies have shown that patients whose seizures were under control showed higher levels of cognitive function than those whose epilepsy was drug-resistant (22, 23). Therefore, we examined whether increasing parenting stress for parents of children with DRE was due to children’s epilepsy-specific characteristics but not children’s high-severity cognitive dysfunction. In our study, after controlling for children’s cognitive abilities, the parents of children with DRE also reported higher parental stress than those of children with CE. Overall, we found that seizure control was an important predictor of parenting stress after considering the cognitive function of children with epilepsy.

Children with epilepsy exhibit more mental health problems than those in the normal population (24) and are more likely to externalize problems rather than internalize problems (25). These factors interact dynamically and synergistically to yield a complex network of forces that may prevent children from reaching their full potential. Furthermore, children with combined epilepsy and impaired cognitive function often exacerbate their emotional, psychological, and academic difficulties that can increase parenting stress (26). Overall, comorbid psychopathology in children with DRE significantly increases parental stress, further affecting the children. Therefore, assessing comprehensive mental health in parents of children with DRE and early intervention may improve the mental healthcare of parents of children with high-severity epilepsy (9).

Parenting stress in parents of children with epilepsy is related to their children’s epilepsy severity. Specifically, the significant finding of this study was that parental stress levels were significantly related to the children’s epilepsy-specific characteristics. Children with DRE often present epilepsy-specific developmental problems that contribute to increased parental stress (27). High levels of parental stress can be explained by parents’ concerns about seizure recurrence, possible adverse effects of anticonvulsants, social stigma, and lifestyle consequences of the disease (28). Future studies to further determine the association between these epilepsy-specific factors and the parenting stress of parents of children with epilepsy may be warranted.

## Limitations

This study had several limitations. First, it was limited by the inclusion of participants from a medical center in a metropolitan region with high recruitment rates. This inclusion potentially limits the generalizability of our findings to children with epilepsy in rural areas as the available resources to decrease parenting stress among parents of children with epilepsy may be poor in such areas. Second, the study did not allow for the analysis of other variables such as etiology, type of epilepsy, and types of anticonvulsants. These epilepsy-specific factors are intricately intertwined and have further significant impact on parents of school-age children with DRE. Third, this was a cross-sectional study that lacked longitudinal follow-up of parenting stress as treatment progressed.

## Conclusion

Our analyses increase the understanding of parenting stress among parents of school-age children with epilepsy, highlighting the finding that parents’ parenting stress levels are associated with the severity of their children’s epilepsy. Our findings may inform the development of evidence-based interventions for reducing parenting stress in caregivers of pediatric patients with epilepsy, especially for children with drug-resistant epilepsy.

## Data availability statement

The original contributions presented in this study are included in the article/supplementary material, further inquiries can be directed to the corresponding author.

## Ethics statement

The Institutional Review Board of the Chung Shan Medical University Hospital approved the study and the parents of all the participants provided informed consent (CS2-20155).

## Author contributions

H-HL conceived and designed the experiments, analyses, and revised the draft. C-YT and I-CC contributed to the data collection and analyses. J-DT authored or reviewed drafts of the manuscript and approved the final draft. All authors contributed to the article and approved the submitted version.
